# American Medical Association

**Published:** 1884-07

**Authors:** Jno. S. Marshall


(TO BE CONTINUED.)

---

### AMERICAN MEDICAL ASSOCIATION.

### SECTION OF ORAL AND DENTAL SURGERY.

### SESSION OF MAY 7, 1884.

REPORTED FOR THE INDEPENDENT PRACTITIONER BY JNO. S. MARSHALL, M.D.

The section was called to order at 2.30 P. M., by the chairman.

The minutes of the last meeting were read and approved.

Dr. Williams moved the appointment of a committee of three to draft suitable resolutions upon the death of Prof. Samuel D. Gross, of Philadelphia. Carried.

Dr. G. V. Black made a motion to add the chairman of the section to the committee. Carried.

The chairman appointed as such committee, Drs. Williams, Allport, Friedrichs and Brophy.

A volunteer paper was then read on

DENTAL CARIES AND ITS RELATIONS TO THE GERM THEORY OF DISEASE; BY G. V. BLACK, M. D., D. D. S., JACKSONVILLE, ILL.

Dr. Black opened his paper with a description of the clinical aspects of dental caries, as follows:

"Caries of the teeth has been defined as a molecular disintegration of the tooth's substance, or a breaking down of the chemical constituents of the tooth, molecule by molecule. This destruction always has its beginnings on the surface of the tooth, or in some pit, crevice, or other imperfection in the enamel, and it spreads from this point as a focus in every direction, the dentine being destroyed more rapidly than the enamel. Hence, it usually happens that the cavity is larger within, than on the surface of the tooth.

"Caries does not seem to be a simple solution of the tooth substance; sometimes we find nearly all of the material removed from the cavity; in other cases we find the dentine reduced to a pulpy or semi-gelatinous mass, in which the structure of the dentine is more or less perfectly preserved.

"Some decays are white, some are black, some have a yellow tinge. All the shades from white to black may be found."

He then stated it was his intention to confine himself as closely as possible to the discussion of the probable cause of caries. "I say *probable* cause, for I do not assume that the cause or causes are certainly known. Indeed, we may say that at the present time there is the greatest disagreement among even the best informed men on this important subject, and that it is still very uncertain that any of the theories in regard to the matter are correct." He recognized the fact, however, "that more than one of these explained the phenomena with sufficient accuracy to be of great value, both in the prevention and treatment of the affection." In reviewing the various theories, he said the acid theory had formed a good working basis upon which to found the principles of practice, but that from a scientific standpoint there are many objections to it. "According to the chemical theory, the substance of the tooth is decomposed by an acid; this acid acts more readily on dentine than upon the enamel, therefore the tendency to the enlargement of the cavity toward the internal portions of the tooth."

Attempts have been made by Dr. Watt and others in America, to define the acids acting upon the teeth, and to classify the varieties of decay caused by these acids—nitric, causing white decay; sulphuric, black decay; and chlorohydric, the intermediate colors. In Europe the views of Dr. Watt are not endorsed, for the acids causing the affection are believed to be the lactic, acetic, and similar acids, belonging to the organic group.

Magitot, of Paris, also claimed, after extended experimentation in this direction, that decay is caused by acids — lactic, acetic, butyric, etc.— and that these acids are derived from the saliva, as a product of fermentation. Magitot admits also the agency of micro-organisms in the production of these acids.

Leber and Rottenstein attempted to prove the causes of dental caries to be the action of acids, and the rapid development of a parasitic plant—the *leptothrix buccalis.* They do not claim that this fungus is capable of penetrating normal tooth structure, but when the surfaces have been attacked by acids and become softened, the micro-organism may enter such breaks in the continuity and continue the work of destruction, but how this is accomplished is left without explanation.

Dr. Black then said: "We can conceive, however, that they may do something to assist the softening process by the out-pouring of a digestive fluid. If, however, this fungus gave a fluid that could digest a tooth, we would think that sound teeth should be very scarce, for it grows abundantly in every mouth."

He next deals with the later experiments of Dr. Miller, of Berlin, and said: "They bear the stamp of being more carefully performed than any that have previously come to our knowledge." "Dr. Miller begins the series of articles in the INDEPENDENT PRACTITIONER with this sentence: 'During the last two years I have stated at different times and places that the first stages of dental caries consist in a decalcification of the tissues of the teeth by acids, which are for the greater part generated in the mouth by fermentation. The object of the investigations described in this and following papers is to determine this ferment, and the conditions essential to its action.'" Dr. Miller's experiments proved: *First*, that the ptyaline of the saliva could not so change starch as to produce an acid. The starch was changed to sugar, but the reaction went no further, the solution remaining sweet indefinitely, if precautions were taken to guard against the admission of germs. This proved the "acidifying power" to come from some outside source.

*Second*, That the cause of the change in the starch from sugar to an acid was an organized ferment, capable of propagating itself. This was proved by the infection of sterilized culture fluids with carious dentine.

*Third*, That the organized ferment was a micro-organism (*bacterium lactis*) capable of producing lactic acid.

*Fourth*, That the acid, in his infected culture fluids, is formed in sufficient quantity to decalcify sections of dentine, while in those not infected with the micro-organisms the sections remained unchanged. Dr. Black then said: "This, when compared with the best experimentation previously made, marks a great advance. One point seems to have been gained. One organism has been traced thus far, and may now be said to have been proven to be able to produce certain of the phenomena of decay. But this is not all. There is much yet to be done. True, one other point is spoken of by Dr. Miller. All who have made a careful study of caries know that there is a peculiar enlargement of the tubules, which is not seen in dentine softened by acids alone. Dr. Miller has been looking for this also, and not without success; for in some sections of dentine exposed to the action of the cultures, he found the organisms crowding into the tubules, and tells us that he also found them enlarged, as in natural caries of the teeth in the mouth; indeed, that he had before him veritable caries, artificially produced. * * * There is nothing in these experiments that is not in harmony with known facts, unless it be the widening of the tubules by the crowding in of the organisms. It is known by previous experiments that this widening is not caused by the lactic acid as it exists dissolved in the surrounding medium, and I think very few will be willing to concede that the organisms can accomplish this by physical force. This point requires further investigation, and its study will doubtless lead to further discoveries. * * * Now this widening of the tubules is a conceded fact. It is also shown that it is not done by the lactic acid, nor can it be done by the physical force of the organisms, but it can, in all probability, be done by the digestive fluid of the organism."

Dr. Black believes that there will be other organisms discovered capable of producing dental caries, viz: the organism of the butyric fermentation, possibly of the acetic, and a large number of other acid fermentations, and thinks also that it may be caused by other vital processes than the acid fermentation.

In explaining the *modus operandi* of the *bacterium lactis* in causing decay, he says: "I have repeatedly said that the waste products of an organism prevented the activity of that organism, when collected in a certain amount. How, then, can this organism continue to thrive in its own waste product, and thus continuously promote caries by furnishing more, and still more, of this waste product? Simply enough. Every chemist who has studied lactic fermentation has been in the habit of introducing some form of lime into the fermenting fluid, to "fix" the lactic acid, in the form of a lactate of lime, in which case it does not hinder the progress of the fermentation. * * * Now, in the production of caries, the tooth presents the lime for the formation of the lactate, and thus furnishes the very conditions necessary to the continuous growth of the organism."

He also called attention to another class of phenomena which may possibly participate in the production of caries, viz.: Tissues otherwise normal, when under certain forms of inflammation throw out an irritating acid fluid, producing excoriation of the skin, etc. Dead bone, ivory driven into the flesh, sponge, cat-gut ligature, etc., are dissolved and removed. The query is then raised whether a substance may not be formed in the same way in the mouth, and produce the phenomena of caries.

Of this he said he was thoroughly convinced, years ago, but attempted to explain it as being caused by the formation of an acid.

"Soluble ferments do not seem to depend for their action on either acidity or alkalinity. They seem to be controlled by some other than the known chemical laws, and their action is not yet understood. We have no means of explaining them. If a piece of ivory thrust into the flesh is attacked and burrowed out by a secretion thrown out by virtue of the irritation induced, why may not a tooth be attacked in the same way, by virtue of an irritation of the tissues about the neck, or during the irritation consequent upon its eruption, when this is unusually prolonged? As a matter of fact, it has been observed that decays are prone to occur in just such situations as tend to confirm this hypothesis."

He claimed that this was one of the "first steps," though not the only one, in the initiation of decays, by which other forces coming later are enabled to get a foot-hold, and that this effect is produced only when "the tissues are in contact or very close proximity," because if otherwise the secretion would be lost in the fluids of the mouth before it could have time to produce its effects upon the tooth structure. The teeth most often affected are the wisdom teeth, that erupt very slowly, the buccal surface of the molars generally, and the labial surfaces of the superior incisors. If noted early in its progress, it will be found that it differs materially from other forms of decay, in that it always has its beginnings under the free margins of the gums, produces no visual change in the appearance of the tooth, except that it is rather whitish, but on probing a cavity of slight depth is disclosed. Often it happens that the enamel is so softened that it can be scraped away over a considerable surface, sometimes extending into the dentine.

Occasionally these characteristics are manifested in the crowns of the wisdom teeth, and sometimes in the first molars, when eruption has been slow and the gums in a state of chronic irritation for a long period. Imperfections in structure are not necessary to prepare the way for its manifestations, for it as often appears upon the smooth surfaces as in pits or crevices.

Those cases of decay occurring in persons of middle life or older, and sometimes in young persons, which have their seat at the junction of the enamel and cementum, are many times of the same character, having their beginning in some irritation of the gum at the immediate spot. If these are closely examined early in their history, "it will be found that the cementum has been removed, and the enamel has become chalky; soon after this, if the case continues to progress, the gum becomes everted so as to expose the breach in the tooth," and it is many times exquisitely sensitive. After a few weeks, or months, the sensitiveness may cease, and then it will be discovered that the case has taken on the characteristic features of caries. In other cases progress is arrested, and it assumes a dark color, or it may run a rapid course, maintaining an ashy tint.

The paper closed with the opinion that this form of decay was caused by a soluble ferment, produced at that point by the irritation to the tissues in contact with the tooth, in precisely the same way that a chronic irritation of the peridental membrane causes a partial absorption of the root of a permanent tooth, and that the very common cases of decay occurring under plates at the free margins of the gums have their beginnings in the same way, the rapid progress of such cases being due to the evertion of the free edge of the gum, and the formation of a pocket by the aid of the plate, in which fermentation progresses under the most favorable circumstances.

### DISCUSSION.

*Dr. Marshall.*—In Dr. Miller's experiments on dental caries, whether produced in the mouth or artificially in his culture fluids, he invariably found an enlargement of the dental tubuli to the extent of the penetration of the affection, and that the tubuli were filled with micro-organisms.

Dr. Bödecker, of New York, found the same enlargement of the tubuli, but minus the micro-organisms, in his experiments with regard to the action of arsenious acid upon healthy dentinal tissue.

Dr. Black refers the cause of the enlargement in Dr. Miller's experiments under both conditions to the action of a soluble ferment produced, as a waste product, by the *bacterium lactis,* and which dissolves or digests the organic portion of the tubuli, and the inorganic material surrounding it, while Dr. Bödecker refers the condition found in his experiments to inflammatory action, resulting in absorption caused by the irritation of the arsenious acid.

I would like to ask Dr. Black whether he considers the conditions found by these gentlemen to be identical.

*Dr. Black*—I will first say that I do not regard the results as caused in both cases by the *bacterium lactis,* but in both cases the results are brought about, in all probability, by a digestive body. The action in each instance is the same in kind. The philosophy of the widening is the same in both instances, the difference being that the digestive bodies that do the work are produced by different forms of life. In the case of Dr. Bödecker's experiments the conditions described could only be due to the formation by the irritated dentinal fibrils of a menstrum which digests and removes the tubular wall. In Dr. Miller's experiments the bacteria form a menstrum which digests and removes the walls of the tubuli.

In the case of the *bacterium lactis,* a digestive body or diastase has now been demonstrated by Dr. Miller. The organism is fully proven to be able to perform the act of digestion, and we know that the tissues of the higher animals have the power of removing parts by what is usually called absorption, and which is a form of digestion.

It has been well shown that the pulp chamber may be enlarged in this way in cases of long-continued irritation of the pulp. Bone is also easily removed under pressure, evidently by a similar vital process, that of digestion by means of a digestive body.

*Dr. Allport.*—Dr. Bödecker's experiments with arsenic upon dentinal tissue (though not yet fully completed), were upon living tissue, and evidently prove that there was inflammatory action set up in the tubules, causing absorption of material. Dr. Miller's experiments were upon dead tissue (teeth out of the mouth), and the action seems to have been a chemical one. In the first, the process was a vital one, and may be in kind like that manifested in the absorption of the roots of deciduous teeth, the removed material being largely taken into the general circulation, which may have been re-moleculized and converted into new tissue, or carried off as a waste product; while in the other case, the action was entirely from without, and would seem to have been from acids, and independent of vital processes within the tissues. I can imagine how micro-organisms may produce a sufficiently exalted irritation of the dentinal fibers to cause absorption of material similar to that produced by the irritation of arsenic in the experiments of Dr. Bödecker. But in either of these conditions I should expect the changed material would be largely carried off in the general circulation, the same as it is in the enlargement of the pulp cavity consequent upon continued irritation of the pulp, as suggested by Dr. Black, or the absorption of the roots of the deciduous teeth. In both cases the organic and inorganic materials are removed entire, while in caries occurring in the mouth, the inorganic material only is largely removed, the organic portion remaining behind to die, be decomposed, and washed away, or pass off in gases. In the experiments of Dr. Miller, the action is external and chemical, resulting in a simple change of the elements, and the removal consequent upon the affinity of lime for acids. Whether the fungi excrete an acid or not (and more than likely it does), is of no material consequence, so long as the acid is found there. It is the affinity of lime for acids, no matter how produced, whether from a decomposition of foreign matter or excreted by the fungi, that, to my mind, produces the changed condition in the devitalized dentine. I have, of course, been much interested in the arguments and experiments of Drs. Black and Miller. They are highly interesting, and there is little doubt that we are approaching a correct solution of this vexed question, but it will be well to pause before we pronounce too confidently that either of these gentlemen accounts for all the phenomena of dental caries.

*Dr. Williams.*—May not the enlargement of the tubules be caused by the physical force of the micro-organisms exerted upon the walls of the tubules? In the vegetable kingdom such instances are not of rare occurrence. Rocks have been split, and large masses of solid material moved by the continued pressure of fungous growths.

*Dr. Friedrichs.*—Milles and Underwood first called attention to the enlargement of the dentinal tubuli, and they presented microscopical specimens to substantiate their views. Dr. Bödecker's experiments do not prove anything. I would not put much reliance upon the observations of *two* experimenters. I think we are begging the question. Some observers can see whatever they desire to see, and later, their observations are proved to be worthless. May not the enlarged appearance of the tubuli be due to the methods used in preparing the slides?

*Dr. Allport.*—Would suggest that Dr. J. L. Suesserott, of Chambersburg, Penn., be invited to speak upon this subject.

*Dr. Suesserott.*—I am certainly very much pleased with the paper just read by Dr. Black. It proves the truth of the lines by Butler, that—

> * Large fleas have little fleas,
> And these have fleas to bite 'em;
> And there are fleas to feed on these,
> And so *ad infinitum.*

Various remedies have been introduced for the antiseptic treatment of surgical diseases, the most potent of which is bichloride of mercury. As used in general surgery, a solution of 1 to 1,000 is perfectly harmless, and I do not see why it might not be used with safety and success in the treatment of dental caries.

*Dr. Harlan.*—The *bacillus anthrax* and the *bacillus tuberculosis* can live in absolute alcohol, and also in a five per cent. solution of chloride of zinc. In order to know how to destroy micro-organisms, we must study the individual organism, and apply the special agent which will destroy their vitality. Eugenol, in the strength of 1 in 75,000, will destroy bacteria, and a weak solution will prevent the development of the spores.

*Dr. Black.*—I quoted an expression of Dr. Miller, saying it was unfortunate, viz: "the first stages of dental caries consist in a

* "So, naturalists observe, a flea
Has smaller fleas that on him prey;
And these have smaller still to bite 'em,
And so proceed *ad infinitum.*"—*Jonathan Swift.*

decalcification of the tissues of the teeth by acids, which are for the greater part generated in the mouth by fermentation." This is the same question discussed of old, of who led the pig to market, the man or the string. The string is the means used by the man, and the acid is the means used by the micro-organisms, but the acid can no more cause decay, or even exist in that position without the organisms, than the string can exist and lead the pig without the man. The micro-organisms that produce caries must have free oxygen. Therefore, if you plug a tooth perfectly over a little softened dentine, you exclude the free oxygen, and destroy the organisms. It is possible, of course, that an organism not requiring free oxygen might produce caries, but all the evidence we have is against that supposition.

*Dr. Friedrichs.*—It seems to me we are still begging the question. What is the need of antiseptic treatment of caries if micro-organisms cannot exist without free oxygen? When a cavity of decay is hermetically sealed, no oxygen can enter, and, consequently, micro-organisms cannot develop.

*Dr. Marshall.*—It has been proved by Pasteur, that there are certain forms of micro-organisms that exist without free oxygen; in fact, cannot develop in that medium; and it is not at all improbable that such micro-organisms may exist as factors in the cause of secondary caries.

*Dr. Black*—Closed the discussion, by saying: We must come to the point of finding specific antiseptics for specific micro-organisms. A poison for one form is not necessarily a poison for another. Only about seventeen per cent. of alcohol can be produced by the yeast plant, because the excretory product of the plant—alcohol—in this degree of concentration becomes a poison to the plant, and prevents further growth. This same alcohol, however, is the natural food of the *mycoderma aceti.*

This plant produces acetic acid, which in turn will stop the growth of its plant at a certain degree of concentration. This will now become the food of other organisms. So it is; that which is poisonous to the one is not, necessarily, poisonous to another. Certain diseases occur in the adult. We believe some of these diseases are produced by micro-organisms. There is some difference in the nature of the tissues or fluids in the two cases that renders the adult unfavorable soil for the growth of these organisms. This gives some hope that in the continuous round of research some means may be found of antagonizing micro-organisms without a resort to poison, such as is now done. These will certainly be different for different organisms.

On motion, it was agreed that, owing to the lateness of the hour, the reading of the paper by Dr. Harlan be deferred, and that it be the first order of business at the session to-morrow. The session then adjourned to meet at 2 30 P. M. to-morrow.

(TO BE CONTINUED.)

